# The effect of hysteroscopic endometrial polypectomy combined with LNG-IUS treatment on polyp recurrence: a multicenter retrospective study

**DOI:** 10.3389/fsurg.2025.1557877

**Published:** 2025-03-06

**Authors:** Cheng Peng, Jie Liu, Hongmei Ding, Hui Duan, Lan Chen, Zichang Feng

**Affiliations:** ^1^ Department of Obstetrics and Gynecology, Nanfang Hospital, Southern Medical University, Guangzhou, China; ^2^ Department of Gynecology, YunFu People's Hospital, YunFu, China

**Keywords:** hysteroscopic polypectomy combined with LNG-IUS, multivariate logistic regression analysis, predictive model, survival analysis, polyp recurrence

## Abstract

**Background:**

Hysteroscopic polypectomy is a common method for treating endometrial polyps, but postoperative recurrence remains a key concern. Recently, the levonorgestrel intrauterine system (LNG-IUS) has been suggested to reduce the recurrence rate of polyps. This study aims to investigate the differences in recurrence rates and recurrence-free survival times between hysteroscopic polypectomy alone and hysteroscopic polypectomy combined with LNG-IUS, and to assess their impact on recurrence.

**Methods:**

This study retrospectively included clinical data from 1,160 patients with endometrial polyps from 2020 to 2021, the follow-up period was 1 year. Patients were divided into two groups based on whether LNG-IUS was used after surgery: the Hysteroscopic Polypectomy group (*n* = 812) and the Hysteroscopic Polypectomy Combined with LNG-IUS group (*n* = 348). Based on the analysis, two multivariate logistic regression models were constructed: the first model included only the factors with significant differences from the statistical analysis, while the second model additionally included the treatment method. The predictive abilities of the two models for polyp recurrence were compared. The recurrence-free survival time differences between the two treatment methods were also analyzed.

**Results:**

Univariate logistic regression analysis showed that birth history, menstrual symptoms, quality of life, and treatment method were significantly associated with polyp recurrence. In multivariate logistic regression models, the second model, which included the treatment method, had a higher AUC (0.703) compared to the first model (AUC = 0.676), which included only significant factors. The recurrence-free survival rate in the group that received hysteroscopic polypectomy combined with LNG-IUS was significantly higher than in the hysteroscopic polypectomy group.

**Conclusion:**

Hysteroscopic polypectomy combined with LNG-IUS significantly reduces polyp recurrence and extends recurrence-free survival time. Multivariate logistic regression analysis indicates that including the treatment method improves the predictive ability of the model, suggesting that combined treatment plays an important role in reducing polyp recurrence and is worthy of further clinical promotion and application.

## 1 Introduction

Endometrial polyps are a common gynecological condition characterized by localized overgrowth of the endometrial tissue, often associated with abnormal uterine bleeding and infertility (1, 2). Although most endometrial polyps are benign, they can still progress to endometrial cancer, particularly in peri- and post-menopausal women (3). For symptomatic polyps or those with malignant potential, hysteroscopic polypectomy is currently considered the most common and effective treatment (4). By removing polyps under direct visualization, this procedure not only alleviates symptoms but also provides tissue for histopathological diagnosis (5). However, despite the effectiveness of hysteroscopic surgery, the issue of polyp recurrence post-surgery remains a significant clinical challenge, affecting long-term outcomes and patient prognosis (6).

Literature reports vary on postoperative recurrence rates, with some studies indicating that recurrence rates may reach as high as 15% to 50% within 1 to 3 years (7, 8). Factors influencing recurrence include the patient's age, hormonal status, polyp size, and number (9). To reduce recurrence, researchers have explored various postoperative interventions. In recent years, the levonorgestrel intrauterine system (LNG-IUS), an intrauterine device that continuously releases low-dose progestin into the endometrium, has gained attention for its potential mechanism in reducing polyp recurrence by inhibiting endometrial hyperplasia and reducing menstrual blood flow (10, 11). LNG-IUS has been widely used in treating conditions such as endometrial hyperplasia and menorrhagia, with some proven efficacy (12). However, clinical studies on the efficacy of combining hysteroscopic polypectomy with LNG-IUS in reducing endometrial polyp recurrence are still limited, and the results remain inconsistent. Therefore, further investigation into the effectiveness of this combined treatment in reducing recurrence is crucial.

This study aims to evaluate, through a retrospective analysis, the differences in polyp recurrence rates and recurrence-free survival times between patients undergoing hysteroscopic polypectomy alone and those receiving hysteroscopic polypectomy combined with LNG-IUS. First, we conducted a statistical analysis of baseline data and menstrual-related indicators for both groups to understand their basic characteristics and differences. We then used multivariate logistic regression analysis to build predictive models, focusing on factors related to polyp recurrence and exploring the predictive ability of the models with and without the inclusion of the treatment method. By comparing the AUC values of the two multivariate logistic regression models, we aimed to further assess the clinical efficacy of LNG-IUS in reducing polyp recurrence and its potential impact on long-term prognosis. Additionally, through survival analysis, we compared the recurrence-free survival times of the two treatment methods, further validating the effect of LNG-IUS in extending recurrence-free survival time.

This study not only provides data to support the clinical assessment of polyp recurrence risk following hysteroscopic polypectomy but also offers theoretical backing for the promotion of the emerging treatment strategy combining LNG-IUS. By constructing predictive models and evaluating their effectiveness, we hope to offer clinical practitioners guidance in managing and treating recurrent endometrial polyps, optimizing treatment plans, and ultimately improving patients' long-term prognosis.

## 2 Materials and methods

### 2.1 Study population

The study data were sourced from the electronic medical record systems of the Department of Gynecology at Southern Medical University Nanfang Hospital and Yunfu People's Hospital between January 2020 and January 2021. Data included patients' baseline characteristics, postoperative follow-up data, and polyp recurrence status. All patients underwent regular follow-up for one year postoperatively, with polyp recurrence monitored via imaging or pathological diagnosis. The inclusion criteria were: patients diagnosed with endometrial polyps and undergoing hysteroscopic polypectomy. Patients were required to have at least 12 months of postoperative follow-up and provide complete clinical data. Exclusion criteria included: (1) preoperative diagnosis of endometrial cancer or other malignant lesions; (2) preoperative hormone therapy or other treatments that could influence polyp recurrence; (3) incomplete follow-up data or loss to follow-up.

### 2.2 Surgical procedure

The hysteroscopic polypectomy is performed 3–5 days after the end of menstruation. 12 h before the procedure, 0.2–0.4 mg of misoprostol tablets are placed in the posterior vaginal fornix. The patient is positioned in the lithotomy position, and a vaginal speculum is placed. Once the cervix is fully exposed, the hysteroscope is inserted into the uterine cavity with a distension pressure setting of 80–100 mmHg. A circular electrode is used to excise the endometrial polyps. After excision, electrocauterization is performed for hemostasis.

For patients receiving combined treatment (Hysteroscopic Polypectomy combined with LNG-IUS), the levonorgestrel intrauterine system (LNG-IUS) is placed in the uterine cavity 4 days after the first menstrual period following the procedure. The system contains 52 mg of levonorgestrel, with a daily release rate of approximately 20 micrograms (µg).

### 2.3 Data collection

Baseline variables collected in this study included age, body mass index (BMI), smoking, drinking, marital status, diabetes, hypertension, birth history, and previous history of uterine surgery. The size and number of polyps. In addition, average menstrual-related indicators from the three months prior to surgery were recorded, including menstrual blood loss (MBL, Obtained using the sanitary pad weighing method), menstrual cycle length, menstrual duration, menstrual symptoms, quality of life (SF-36 score), and endometrial thickness. In this study, polyp recurrence was defined as the reappearance of endometrial polyps confirmed by imaging (e.g., ultrasound) or pathological examination during follow-up. The diagnosis of polyp recurrence was based on the recurrence of symptoms (e.g., abnormal uterine bleeding) during follow-up, confirmed by diagnostic evaluation. If no recurrence occurred during the follow-up period, the case was recorded as non-recurrent.

The Numerical Rating Scale (NRS) was used to assess the severity of symptoms such as menstrual pain. The scale involves patients rating their pain or discomfort intensity on a scale of 0 to 10, with 0 indicating “no pain” or “no discomfort”, 1–3 indicating “mild pain” or “mild discomfort” that is present but tolerable and does not affect daily activities, 4–6 indicating “moderate pain” or “moderate discomfort” that is more noticeable and may affect daily activities but remains bearable, 7–9 indicating “severe pain” or “severe discomfort” that severely impacts daily life and work, and 10 indicating “extreme pain” or “unbearable pain” that may require emergency medical intervention. The SF-36 (Short Form-36 Health Survey) is a commonly used tool for assessing quality of life, measuring the patient's health status and its impact on daily life over the past four weeks (13). It consists of 36 questions covering eight health dimensions and assesses physical, psychological, and social functions. Each dimension is scored from 0 to 100, with higher scores indicating better quality of life.

### 2.4 Statistical analysis

First, statistical analysis of the baseline data and menstrual-related indicators of the two groups was performed to evaluate the differences between them. Continuous variables (e.g., age, BMI, menstrual blood loss) were compared using the Wilcoxon rank-sum test, and categorical variables (e.g., smoking, drinking, marital status) were compared using the chi-square test. Next, a univariate logistic regression analysis was conducted to explore factors associated with polyp recurrence. Independent variables included birth history, menstrual symptoms, SF-36 score, treatment method, and other variables with significant differences. The dependent variable was polyp recurrence (1 = recurrence, 0 = no recurrence).

### 2.5 Multivariate logistic regression model construction

Two models were constructed in the multivariate logistic regression analysis:

Model 1: Included only factors with significant differences from the statistical analysis, to evaluate their independent impact on polyp recurrence.
Model 2: In addition to the factors in Model 1, the treatment method (hysteroscopic polypectomy alone vs. combined with LNG-IUS) was added to assess the impact of treatment on polyp recurrence.

### 2.6 Survival analysis

To evaluate the effect of the two treatment methods on recurrence-free survival time, Kaplan–Meier survival analysis was used. Recurrence-free survival time was defined as the time from surgery to either the first polyp recurrence or the last follow-up if no recurrence occurred, measured in days. Kaplan–Meier curves were used to compare the differences in recurrence-free survival times between the hysteroscopic polypectomy group and the hysteroscopic polypectomy combined with LNG-IUS group (14). The log-rank test was used to determine the statistical significance of differences between the two groups, with a *P*-value of less than 0.05 considered statistically significant. The survival time for patients without recurrence was set at 365 days, while the survival time for patients with recurrence was the time from treatment completion to the recurrence of the polyp. The average recurrence-free survival time for both treatment methods was calculated.

### 2.7 Statistical software

All data analyses were performed using R software (version 4.4.1). The glm() function was used for univariate and multivariate logistic regression analyses, the survfit() and ggsurvplot() functions were used to plot Kaplan–Meier curves for survival analysis, and the AUC values were calculated using the pROC package.

## 3 Results

### 3.1 Baseline characteristics of patients with endometrial polyps

A total of 1,160 patients were included, comprising 812 patients who underwent hysteroscopic polypectomy alone and 348 patients who received hysteroscopic polypectomy combined with LNG-IUS. The analysis showed significant differences between the two groups in terms of birth history and previous history of uterine surgery. Specifically, 71.55% of patients in the combined LNG-IUS group had a birth history, compared to 61.38% in the hysteroscopic polypectomy alone group (*P* = 4.55E-27). Additionally, 26.29% of patients in the hysteroscopic polypectomy alone group had a previous history of uterine surgery, compared to only 5.46% in the combined LNG-IUS group (*P* = 6.16E-31). This indicates that birth history and previous history of uterine surgery differed significantly between the two groups. The median age of all patients was 38 years (range 17–58 years), and there was no significant difference between the different treatment groups (*P* = 0.786). The median BMI of all patients was 26.2 (range 16.5–35.1), and there was no significant difference in BMI between different treatment groups (*P* = 0.867). 28.28% of patients smoked, with 29.43% in the polypectomy only group and 25.57% in the combined LNG-IUS treatment group. There was no significant difference between the groups (*P* = 0.200). 33.71% of patients have a drinking habit, 34.73% in the polypectomy only group, and 31.32% in the combined LNG-IUS treatment group, with no significant difference between the groups (*P* = 0.278). 54.57% of the patients were married, and there was no significant difference between the groups (*P* = 0.440). 11.47% of patients had diabetes, and there was no significant difference in the proportion of diabetes between the two groups (*P* = 0.159). 26.72% of patients had hypertension, with no significant difference between the groups (*P* = 0.148). The average size of polyps was 13.68 mm (range 6.20–21.50 mm), and there was no significant difference between the two groups (*P* = 0.121). 47.33% of patients have a single polyp, while 52.67% have multiple polyps. In the polypectomy only group, 45.94% of patients had a single polyp, while in the combined LNG-IUS treatment group, 50.57% had a single polyp, with no significant difference between the groups (*P* = 0.165) (Table 1).

**Table 1:** Baseline information of patients with endometrial polyps.

Patient characteristics	All patients (*n* = 1,160)	Hysteroscopic polypectomy (*n* = 812)	Hysteroscopic polypectomy combined with LNG-IUS (*n* = 348)	*P*-value
Age	38 (17–58)	38 (18–56)	38 (17–58)	0.786
BMI	26.2 (16.5–35.1)	26.2 (16.5–35.1)	26.5 (16.6–35)	0.867
Smoking				0.2003907
Yes	328 (28.28%)	239 (29.43%)	89 (25.57%)	
No	832 (71.72%)	573 (70.57%)	259 (74.43%)	
Drinking				0.2784483
Yes	391 (33.71%)	282 (34.73%)	109 (31.32%)	
No	769 (66.29%)	530 (65.27%)	239 (68.68%)	
Marital status				0.4407394
Married	633 (54.57%)	437 (53.82%)	196 (56.32%)	
Unmarried	527 (45.43%)	375 (46.18%)	152 (43.68%)	
Diabetes				0.1598393
Yes	133 (11.47%)	86 (10.59%)	47 (13.51%)	
No	1,027 (88.53%)	726 (89.41%)	301 (86.49%)	
Hypertension				0.1484746
Yes	310 (26.72%)	207 (25.49%)	103 (29.6%)	
No	850 (73.28%)	605 (74.51%)	245 (70.4%)	
Birth history				4.55E-27
Yes	712 (61.38%)	581 (71.55%)	131 (37.64%)	
No	448 (38.62%)	231 (28.45%)	217 (62.36%)	
Previous history of uterine surgery				6.16E-31
Yes	305 (26.29%)	286 (35.22%)	19 (5.46%)	
No	855 (73.71%)	526 (64.78%)	329 (94.54%)	
Polyps size (mm)	13.68 (6.20–21.50)	13.53 (6.20–21.50)	14.37 (6.40–21.50)	0.121
Number of polyps				0.1657731
Single polyp	549 (47.33%)	373 (45.94%)	176 (50.57%)	
Multiple polyps	611 (52.67%)	439 (54.06%)	172 (49.43%)	

### 3.2 Clinical characteristics of patients with endometrial polyps

The results show that there were no significant differences between the two groups in most indicators, including menstrual cycle length (32.5 days vs. 37 days, *P* = 0.054), menstrual duration (5.5 days vs. 3 days, *P* = 0.241), menstrual symptoms (3 vs. 2, *P* = 0.0603), SF-36 score (77 vs. 82, *P* = 0.1449), and endometrial thickness (6.44 mm vs. 4.82 mm, *P* = 0.3393). However, menstrual blood loss was significantly higher in the hysteroscopic polypectomy alone group compared to the combined LNG-IUS group (44.8 ml vs. 20.7 ml, *P* = 0.0396). Regarding polyp recurrence, 13.55% of patients in the hysteroscopic polypectomy alone group experienced recurrence, whereas only 5.17% of patients in the combined treatment group had recurrence (*P* = 0.000047), indicating a significant advantage of the combined LNG-IUS treatment in reducing polyp recurrence (Table 2). This suggests that the two groups were similar in most preoperative baseline characteristics, while the combined treatment demonstrated a significant advantage in reducing postoperative polyp recurrence.

**Table 2:** Comparison of clinical outcomes between hysteroscopic polypectomy alone and hysteroscopic polypectomy combined with LNG-IUS.

Clinical outcomes	All patients(*n* = 1,160)	Hysteroscopic polypectomy (*n* = 812)	Hysteroscopic polypectomy combined with LNG-IUS (*n* = 348)	*P*-value
Menstrual Blood Loss (ml/cycle)	33.6 (10.7–77.4)	44.8 (30.7–77.4)	20.7 (10.8–46.9)	0.0396
Menstrual Cycle Length (day)	35 (24–42)	32.5 (24–38)	37 (28–42)	0.054
Menstrual Duration	6.5 (3–9.5)	5.5 (3–8)	3 (3–9.5)	0.241
Menstrual Symptoms	3 (0–6)	3 (0–6)	2 (0–5)	0.0603
SF-36	78 (60–95)	77 (60–76)	82 (65–95)	0.1449
Endometrial thickness (mm)	5.15 (1.4–11.3)	6.44 (2.7–11.2)	4.82 (1.4–9.3)	0.3393
Polyp recurrence				0.000047
Yes	128 (11.03%)	110 (13.55%)	18 (5.17%)	
No	1,032 (88.97%)	702 (86.45%)	330 (94.83%)	

### 3.3 Univariate logistic regression analysis for predicting polyp recurrence

The results showed that birth history, menstrual symptoms, quality of life score, and treatment method (hysteroscopic polypectomy combined with LNG-IUS) were significant predictors of polyp recurrence. Specifically, having a birth history (Coefficient = −1.032, *P* < 0.001) and higher quality of life scores (Coefficient = −0.030, *P* < 0.001) were negatively correlated with polyp recurrence, while more severe menstrual pain symptoms (Coefficient = 0.109, *P* = 0.011) increased the likelihood of recurrence. The larger the polyp, the higher the likelihood of recurrence (Coefficient = 0.055, *P* = 0.001). The greater the number of polyps, the higher the likelihood of recurrence (Coefficient = 0.389, *P* = 0.012). The treatment method (Coefficient = −1.770, *P* < 0.001) had a significant impact on polyp recurrence, with combined LNG-IUS treatment significantly reducing the recurrence rate. Other variables, such as previous history of uterine surgery, menstrual blood loss, and endometrial thickness, did not show significant effects in this analysis (Table 3).

**Table 3:** Single factor logistic regression model for predicting polyp recurrence.

Factors	Coefficient	Std. error	*Z* value	*P*-value
Birth history (Yes or No)*	−1.032	0.155	−6.665	0.000
Previous history of uterine surgery (Yes or No)	−0.045	0.173	−0.263	0.793
Menstrual Blood Loss	0.003	0.004	0.806	0.420
Menstrual Symptoms*	0.109	0.043	2.548	0.011
SF-36*	−0.030	0.008	−3.920	0.000
Endometrial thickness	−0.020	0.026	−0.788	0.431
Polyps size*	0.055	0.017	3.203	0.001
Number of polyps*	0.389	0.154	2.527	0.012
Treatment (Hysteroscopic Polypectomy vs. Hysteroscopic Polypectomy combined with LNG-IUS)*	−1.770	0.256	−6.929	0.000

* indicates significant independent factors.

### 3.4 Multivariate logistic regression analysis for predicting polyp recurrence

**Figure 1:**
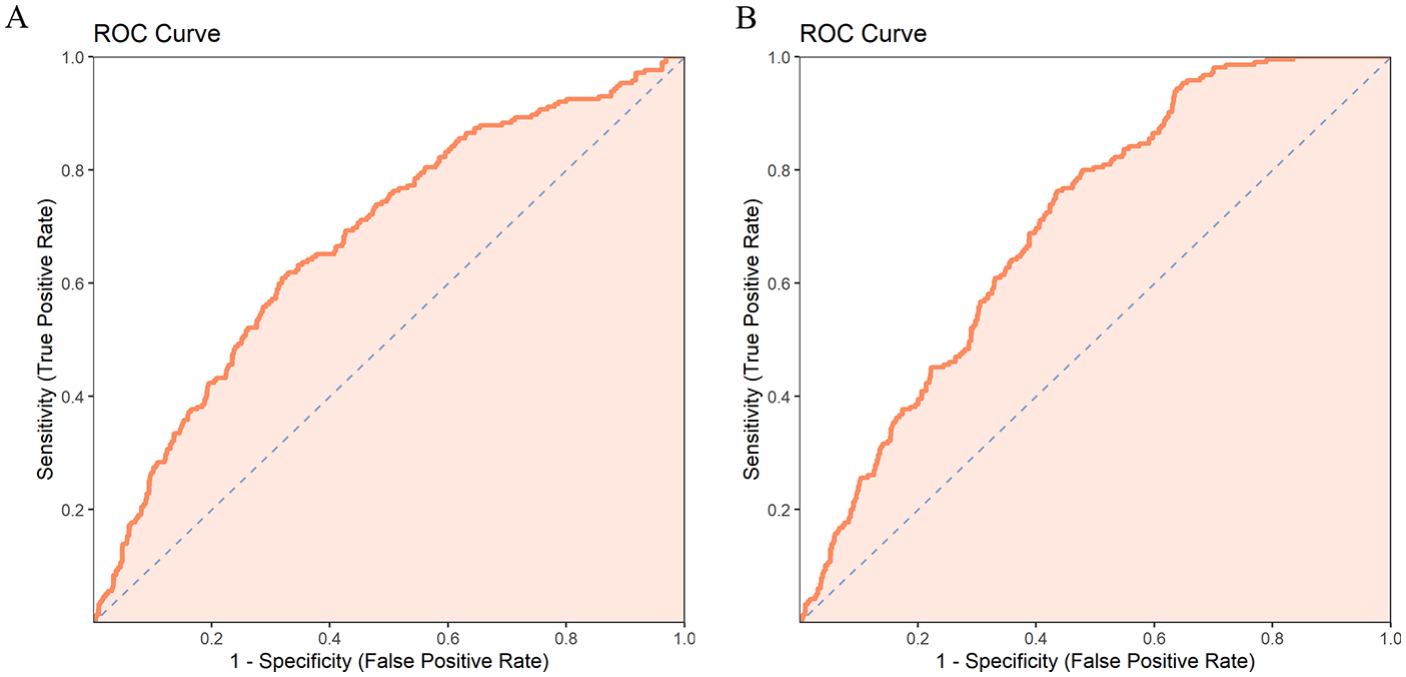
**(A)** ROC curve of model 1. **(B)** ROC curve of model 2.

In Model 1, having a birth history (OR = 0.362, *P* < 0.001) and higher quality of life scores (OR = 0.971, *P* < 0.001) significantly reduced the risk of polyp recurrence, while more severe menstrual pain symptoms increased the risk of recurrence (OR = 1.116, *P* = 0.013). The larger the polyp, the higher the risk of recurrence (OR = 1.055, *P* = 0.003). The greater the number of polyps, the higher the risk of recurrence (OR = 1.637, *P* = 0.002). Other variables, such as previous history of uterine surgery, menstrual blood loss, and endometrial thickness, did not have significant effects on recurrence. In Model 2, which included treatment method in addition to the variables from Model 1, the results showed that combined LNG-IUS treatment significantly reduced the risk of polyp recurrence (OR = 0.228, *P* < 0.001). Birth history, menstrual symptoms, and quality of life score remained significant factors influencing recurrence in this model (Table 4). The ROC curve indicated that the multivariate logistic regression model that included treatment method had stronger predictive power for polyp recurrence (Table 5) (Figure 1).

**Table 4:** Multivariate logistic regression model for predicting polyp recurrence in patients.

Prediction models	B	Std. error	Statistic	*P*-value	OR	CI-lower	CI-upper
Model 1							
Birth history (Yes or No)*	−1.013	0.157	−6.447	0.000	0.363	0.267	0.494
Previous history of uterine surgery (Yes or No)	−0.041	0.179	−0.230	0.818	0.960	0.675	1.364
Menstrual Blood Loss	0.002	0.004	0.382	0.702	1.002	0.993	1.010
Menstrual Symptoms*	0.109	0.045	2.455	0.013	1.116	1.022	1.217
SF 36*	−0.030	0.008	−3.717	0.000	0.971	0.956	0.986
Endometrial thickness	−0.021	0.027	−0.775	0.438	0.979	0.929	1.032
Polyps size*	0.053	0.018	3.017	0.003	1.055	1.019	1.092
Number of polyps*	0.493	0.161	3.064	0.002	1.637	1.194	2.243
Model 2							
Birth history (Yes or No)*	−0.571	0.168	−3.399	0.001	0.565	0.407	0.785
Previous history of uterine surgery (Yes or No)	−0.031	0.182	−0.168	0.866	0.970	0.679	1.385
Menstrual Blood Loss	0.001	0.004	0.137	0.891	1.001	0.992	1.009
Menstrual Symptoms*	0.104	0.045	2.314	0.021	1.109	1.016	1.211
SF 36*	−0.030	0.008	−3.739	0.000	0.970	0.955	0.986
Endometrial thickness	−0.026	0.027	−0.942	0.346	0.975	0.924	1.028
Polyps size*	0.055	0.018	3.044	0.002	1.056	1.020	1.094
Number of polyps*	0.521	0.163	3.202	0.001	1.684	1.224	2.317
Treatment (Hysteroscopic Polypectomy vs Hysteroscopic Polypectomy combined with LNG-IUS)*	−1.480	0.272	−5.445	0.000	0.228	0.134	0.388

* indicates significant independent factors.

**Table 5:** ROC parameters of two prediction models.

Prediction models	AUC	AUC-CI-lower	AUC-CI-upper	Threshold	Youden	Sensitivity	Specificity
Model 1	0.676	0.637	0.716	0.195	0.290	0.609	0.680
Model 2	0.703	0.669	0.737	0.189	0.328	0.763	0.565

### 3.5 Recurrence-free survival analysis

**Figure 2:**
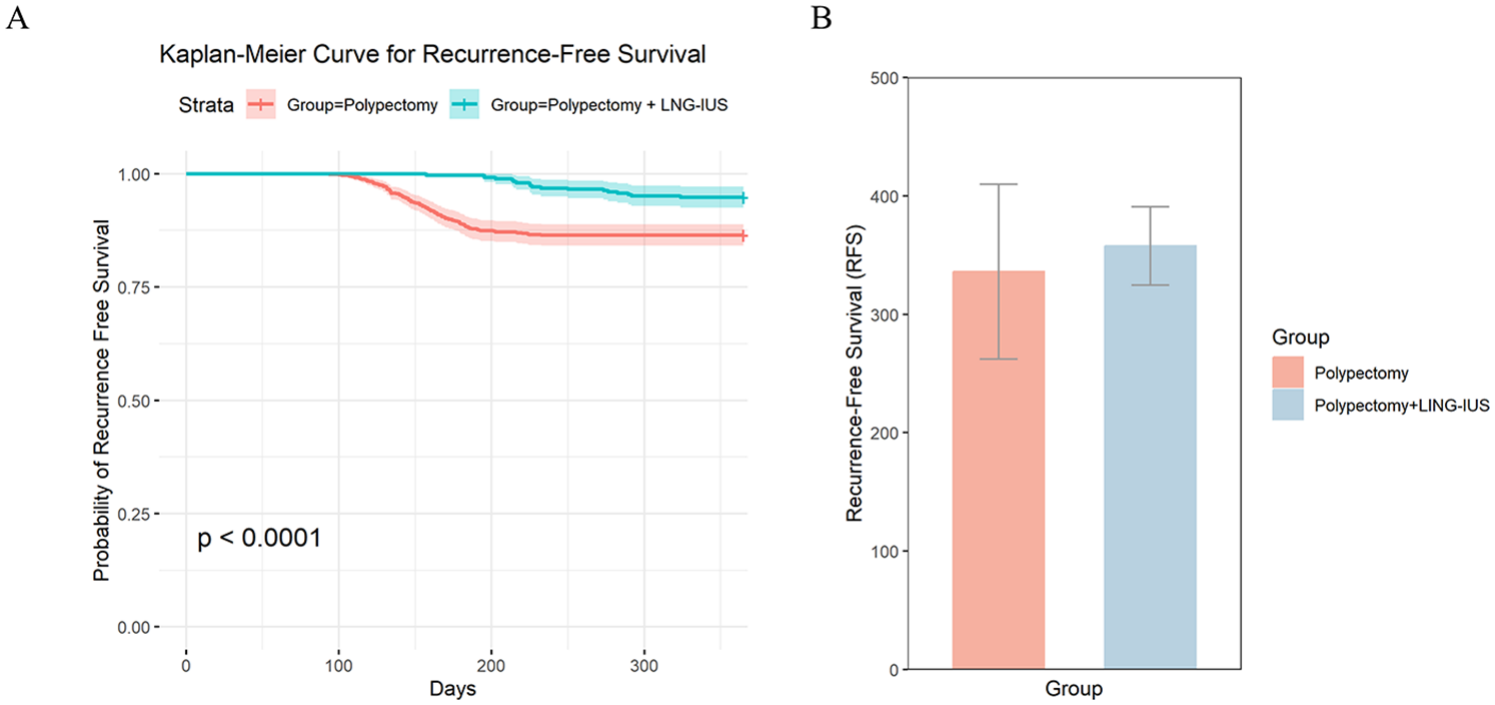
**(A)** Kaplan Meier survival curve plot of recurrence free survival rate. **(B)** The difference in the average recurrence-free survival time between the Hysteroscopic Polypectomy group and the Hysteroscopic Polypectomy combined with LNG-IUS group.

The results showed that the recurrence-free survival rate in the combined LNG-IUS treatment group was significantly higher than that in the hysteroscopic polypectomy alone group, and the curve declined more slowly, indicating a lower recurrence rate. The log-rank test yielded a *P* value of less than 0.0001, indicating a highly significant statistical difference in survival between the two groups (Figure 2A). The average recurrence-free survival time for the Hysteroscopic Polypectomy combined with LNG-IUS group was 357.54 days, while the average recurrence-free survival time for the Hysteroscopic Polypectomy group was 336.06 days. This shows that the combination treatment extended the recurrence-free survival time by 21.48 days (Figure 2B). This suggests that the combined LNG-IUS treatment can significantly extend recurrence-free survival time and reduce the risk of polyp recurrence.

### 3.6 Stratified analysis

**Figure 3:**
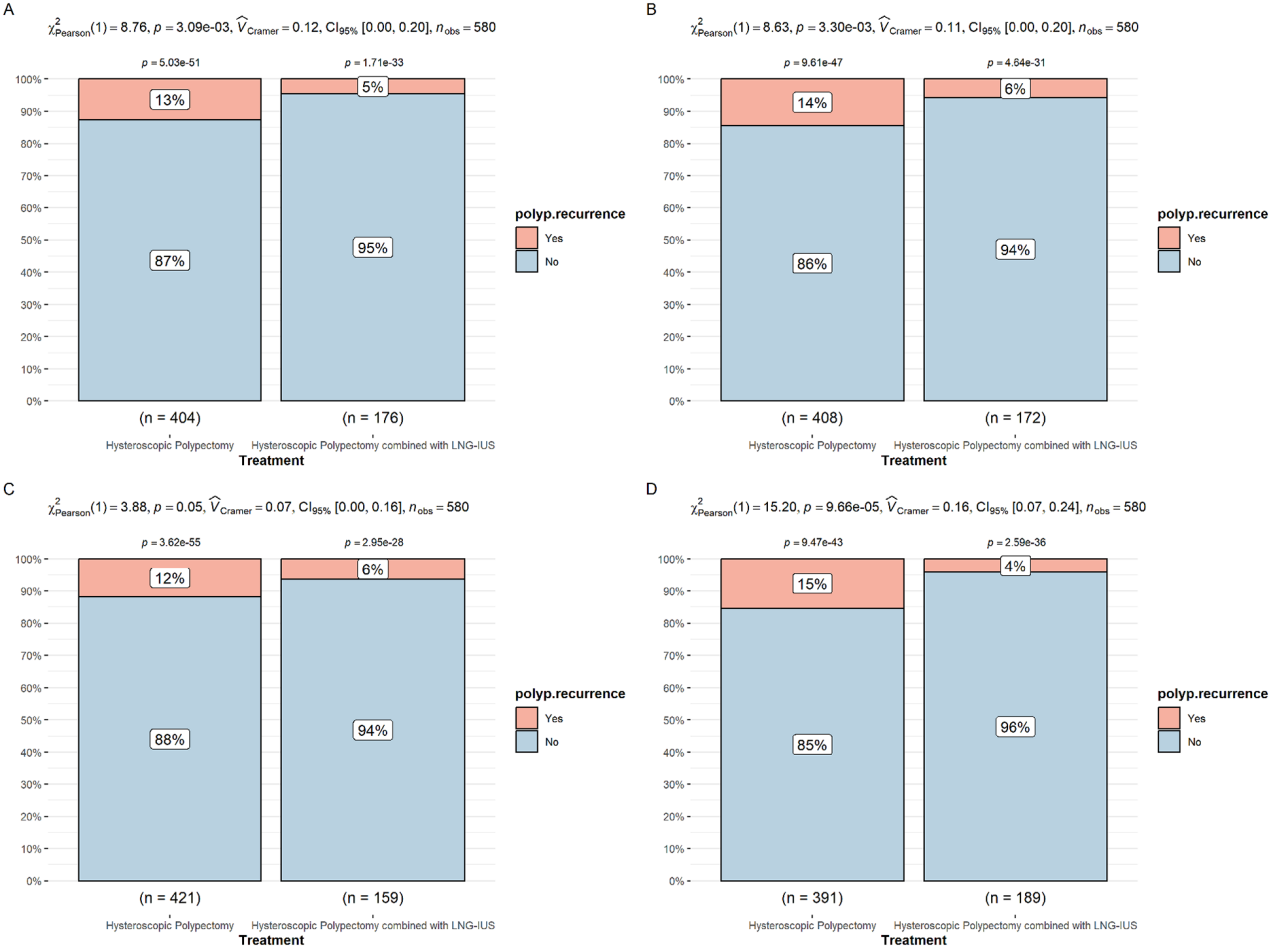
**(A)** Difference in polyp recurrence rates between the two treatment methods in younger patients. **(B)** Difference in polyp recurrence rates between the two treatment methods in older patients. **(C)** Difference in polyp recurrence rates between the two treatment methods in patients with smaller polyps. **(D)** Difference in polyp recurrence rates between the two treatment methods in patients with larger polyps.

Patients older than the median age were classified as older patients, while those younger than the median age were classified as younger patients. Patients with a polyp diameter larger than the median were classified as having larger polyps, and those with smaller polyps were classified accordingly. We further investigated the differences in polyp recurrence rates between the two treatment methods in patients of different ages and with different polyp sizes. The results showed that the recurrence rate in the combined treatment group was significantly lower than in the polypectomy-only group in both younger and older patients, with similar significance between the two age groups, indicating that the lower recurrence advantage of the combined treatment is not age-dependent (Figures 3A,B). The recurrence rate in the combined treatment group was significantly lower than in the polypectomy-only group in both patients with smaller and larger polyps, with a more significant difference in patients with larger polyps, indicating that the combined treatment is more effective in patients with larger polyps (Figures 3C,D).

## 4 Discussion

This study compared the impact of hysteroscopic polypectomy alone and hysteroscopic polypectomy combined with LNG-IUS treatment on endometrial polyp recurrence, revealing that the combined treatment significantly reduced recurrence rates. This finding was further confirmed through multivariate logistic regression analysis and Kaplan–Meier survival analysis. The following discussion focuses on our key findings and compares them with existing literature.

Through multivariate logistic regression analysis, we identified birth history, menstrual symptoms, quality of life score (SF-36), and treatment method as significant factors influencing polyp recurrence. In our model, birth history had a significant negative effect on polyp recurrence, indicating that patients with a history of childbirth had a lower risk of recurrence. This is consistent with previous studies that suggest birth history may reduce the likelihood of polyp recurrence through changes in the endometrium. However, previous history of uterine surgery did not show a significant effect in our analysis, despite some studies suggesting that uterine surgeries may alter endometrial structure and influence polyp recurrence.

Menstrual pain symptoms were shown to be a significant positive predictor of polyp recurrence in our study, meaning that the more severe the symptoms, the higher the likelihood of recurrence (15). This finding has important clinical implications, suggesting that menstrual pain is not just a subjective discomfort but may also reflect underlying pathological changes, potentially serving as an early warning sign of polyp recurrence. Therefore, menstrual pain should be considered a potential indicator for recurrence, and patients reporting persistent or worsening symptoms during follow-up should receive timely imaging examinations or other diagnostic measures to prevent further recurrence.

Additionally, higher quality of life scores (SF-36) were associated with a lower risk of polyp recurrence, indicating that better quality of life may be linked to reduced recurrence risk. This may be because patients with higher quality of life are generally in better physical and psychological health, which may contribute to improved recovery and disease resistance (16). This finding aligns with modern medical perspectives that emphasize the importance of overall health and psychological well-being in disease management and recovery (17, 18). Patients with higher quality of life may also adhere better to postoperative instructions, attend follow-ups regularly, and manage their health more effectively, all of which could help reduce the chance of recurrence. Thus, improving patients' quality of life not only enhances their satisfaction but may also play a role in preventing disease recurrence.

One of the key findings of this study was the significant impact of the treatment method on polyp recurrence. Combined LNG-IUS treatment significantly reduced the risk of recurrence, and this was further supported by the Kaplan–Meier survival analysis results. The log-rank test (*P* < 0.0001) indicated that the recurrence-free survival rate was significantly higher in the combined LNG-IUS group compared to the hysteroscopic polypectomy alone group. This aligns with other studies on LNG-IUS (19). By locally releasing progestin into the endometrium, LNG-IUS suppresses endometrial hyperplasia, thereby reducing the risk of polyp recurrence. The mechanism may involve local regulation of the endometrium, decreasing postoperative endometrial regeneration and abnormal proliferation, thereby lowering the recurrence rate (20, 21). Notably, combined LNG-IUS treatment not only reduced recurrence rates but also improved patients' quality of life. Our study found that the SF-36 score in the combined treatment group was significantly higher than in the hysteroscopic polypectomy alone group, further supporting the advantages of this treatment in improving overall health outcomes.

Our research suggests that combination therapy may have better efficacy for patients with larger polyps, possibly because larger polyps are more prone to recurrence, and LNG-IUS, through its sustained release of levonorgestrel, can more effectively inhibit endometrial hyperplasia, thereby reducing the risk of recurrence. From a mechanistic perspective, the role of LNG-IUS is not limited to local inhibition of endometrial hyperplasia, but may also have a stabilizing effect on the intrauterine environment through long-term hormone release, reducing the chance of polyp recurrence. For larger polyps, stronger long-term hormone intervention may be needed, while LNG-IUS can continuously release levonorgestrel, providing a long-term therapeutic effect.

The results of this study have important clinical implications. For patients prone to recurrence after hysteroscopic polypectomy, combined LNG-IUS treatment can effectively reduce recurrence and improve quality of life. Therefore, LNG-IUS, as a simple and effective adjunct therapy, should be widely promoted in clinical practice, particularly for patients at high risk of recurrence (22). Additionally, our findings suggest that menstrual pain symptoms and quality of life should be key observations during follow-up. Severe menstrual pain may indicate an increased risk of recurrence, while a higher quality of life may be associated with a lower risk. Clinicians should take these factors into account when managing postoperative care to provide personalized treatment and follow-up plans for patients.

Despite the significant findings of this study, some limitations remain. First, although this is a multi-center study, ensuring the broad applicability and representativeness of the data, potential confounding factors and information bias inherent in the retrospective design cannot be fully excluded (23). Second, while we controlled for various clinical variables, some unmeasured factors may still influence the results. Furthermore, the follow-up period was relatively short, limiting our ability to assess long-term recurrence and treatment effects. Future studies should extend the follow-up period to further validate the long-term efficacy of combined LNG-IUS treatment.

This study suggests that hysteroscopic polypectomy combined with LNG-IUS treatment can reduce the risk of recurrence of endometrial polyps and prolong recurrence free survival time. However, considering the small number of patients and relatively short follow-up time in this study, this conclusion may have certain limitations. Although this study suggests that the use of local progesterone can help reduce the risk of polyp recurrence, these conclusions need to be confirmed through larger scale and longer duration studies.

## 5 Conclusion

This study shows that hysteroscopic polypectomy combined with LNG-IUS treatment significantly reduces the risk of endometrial polyp recurrence and extends recurrence-free survival time. Birth history, menstrual pain symptoms, and quality of life score (SF-36) are important predictors of recurrence. However, due to the limitations of the patient sample size and follow-up duration in this study, large-scale prospective randomized controlled trials are needed in the future to further validate the conclusions of this study.

## Data Availability

The original contributions presented in the study are included in the article/Supplementary Material, further inquiries can be directed to the corresponding author.
